# Activity in descending dopaminergic neurons represents but is not required for leg movements in the fruit fly *Drosophila*

**DOI:** 10.14814/phy2.12322

**Published:** 2015-03-05

**Authors:** Katherine Tschida, Vikas Bhandawat

**Affiliations:** 1 Department of Biology, Duke UniversityDurham, North Carolina; 2 Duke Institute for Brain SciencesDurham, North Carolina

**Keywords:** Dopamine, *Drosophila*, in vivo electrophysiology, motor control, neuromodulation

## Abstract

Modulatory descending neurons (DNs) that link the brain to body motor circuits, including dopaminergic DNs (DA-DNs), are thought to contribute to the flexible control of behavior. Dopamine elicits locomotor-like outputs and influences neuronal excitability in isolated body motor circuits over tens of seconds to minutes, but it remains unknown how and over what time scale DA-DN activity relates to movement in behaving animals. To address this question, we identified DA-DNs in the *Drosophila* brain and developed an electrophysiological preparation to record and manipulate the activity of these cells during behavior. We find that DA-DN spike rates are rapidly modulated during a subset of leg movements and scale with the total speed of ongoing leg movements, whether occurring spontaneously or in response to stimuli. However, activating DA-DNs does not elicit leg movements in intact flies, nor do acute bidirectional manipulations of DA-DN activity affect the probability or speed of leg movements over a time scale of seconds to minutes. Our findings indicate that in the context of intact descending control, changes in DA-DN activity are not sufficient to influence ongoing leg movements and open the door to studies investigating how these cells interact with other descending and local neuromodulatory inputs to influence body motor output.

## Introduction

Organisms must flexibly modulate their movements to meet a variety of behavioral demands. Because neuromodulators can transform the output of neural circuits through their effects on diverse cellular processes (Marder 2012), they play a particularly important role in the flexible control of behavior. Motor circuits within the body contain central pattern generator networks that coordinate the movements of the different joints that participate in a given behavior, and these networks are subject to neuromodulation from both local sources and descending inputs from the brain. Dopamine plays a well-established role in the regulation of movement in vertebrates and invertebrates, and dopaminergic descending neurons (DA-DNs) that innervate body motor circuits have been identified across phyla (Commissiong and Sedgewick 1975; Björklund and Skagerberg 1979; Hökfelt et al. 1979; Nässel and Elekes 1992; McLean and Fetcho 2004). Although in vivo genetic manipulations of dopaminergic signaling leave no doubt that dopamine can influence movement via its effects on circuits within the brain (Baik et al. 1995; Giros et al. 1996; Xu et al. 1997; Friggi-Grelin et al. 2003; Kume et al. 2005; Lima and Miesenböck 2005; Lebestky et al. 2009; Riemensperger et al., 2011; Alekseyenko et al. 2013), considerably less attention has been paid to the specific role of descending dopaminergic projections in modulating motor circuits and behavior (Sharples et al. 2014).

Several lines of evidence suggest that DA-DNs play an important role in modulating body motor circuits across a range of species. Dopamine receptors are widely distributed in the spinal cord of vertebrates (Zhu et al. 2007) and in the segmental body ganglia of invertebrates (Kim et al. 2003). Both ex vivo and in vitro electrophysiological studies indicate that dopamine can influence the intrinsic properties and excitability of motor neurons (McPherson and Kemnitz 1994; Schotland et al. 1995; Cooper and Neckameyer 1999; Dasari and Cooper 2004; Han et al. 2007; Puhl and Mesce 2008). Furthermore, studies in reduced body motor circuit preparations have shown that application of dopamine receptor agonists elicits fictive motor rhythms, including crawling rhythms in the leech, flight rhythms in the tobacco horn worm moth, and locomotor rhythms in the pond snail (Claassen and Kammer 1986; Tsyganov and Sakharov 2000; Puhl and Mesce 2008). Similarly, administration of dopamine receptor agonists elicits limb movements in spinal mice (Lapointe et al. 2009) and locomotion and grooming in decapitated fruit flies (Yellman et al. 1997), suggesting that descending dopaminergic inputs can promote the activation of body motor circuits. However, two major issues remain unresolved. First, it is unknown how and over what time scale DA-DN activity is related to movement in intact, behaving animals. Second, it remains untested whether selective manipulations of DA-DN activity have any effect on movement in intact animals, in which other descending inputs to body motor circuits remain unperturbed.

Here, we take advantage of the accessibility and manipulability of the *Drosophila* model system to measure how DA-DN activity represents sensory inputs and motor output in behaving fruit flies and to test the hypothesis that DA-DN activity promotes movement. An earlier study found a cluster of four DA-DNs in the blowfly brain, and a similar cluster of neurons was found in *Drosophila* but was not confirmed to be descending (Nässel and Elekes 1992). Using this study as a starting point, we determined that two of these neurons represent the sole source of dopaminergic input from the brain to the body motor circuits in *Drosophila*. We then employed in vivo whole-cell recordings to characterize DA-DN activity in behaving flies and used electrophysiological and genetic methods to test the effects of selective manipulations of DA-DN activity on behavior. We find that DA-DN spike rates are rapidly modulated during certain behavioral categories of leg movements and scale with movement speed. However, increasing DA-DN activity is not sufficient to elicit leg movements, nor did we detect effects of acutely activating or silencing DA-DNs on the probability or speed of leg movements over a time scale of seconds to minutes. Our findings are inconsistent with the idea that changes in DA-DN activity are sufficient to modulate ongoing leg movements and open the door to studies exploring the interactions of DA-DNs with other descending and local neuromodulatory neurons and their role in additional behavioral contexts.

## Materials and Methods

Data were analyzed with standard parametric and nonparametric statistical tests and are reported as mean plus or minus standard error of the mean, unless otherwise noted.

### *Drosophila* stocks

The TH-GAL4 line is a gift from Serge Birman (ESPCI Paris Tech). Flies of the genotype UAS-mCD8::GFP;TH-GAL4 were used for electrophysiological recordings and behavioral measurements. To visualize axonal and dendritic projections of dopaminergic neurons, TH-GAL4 flies were crossed to flies carrying UAS-DenMark;UAS-syt.eGFP (Bloomington stock collection). UAS-mCD8::GFP;TH-GAL4 flies were crossed with UAS-P2X2 flies (Lima and Miesenböck 2005) to activate DA-DNs using ATP application (see below).

### Retrograde labeling of putative descending neurons

Forceps were used to remove the heads from UAS-mCD8::GFP;TH-GAL4 flies. A pipette was used to apply a small volume of 3K MW tetramethylrhodamine-conjugated dextran (Life Technologies, Carlsbad, CA) to the cut neck, and fly heads were then immersed in external saline and left at room temperature for 1 h. Brains were dissected, rinsed in phosphate-buffered saline containing 0.4% Triton X-100 (PBST), fixed for 20 min in 4% paraformaldehyde (PFA) in PBS, and mounted in Vectashield. Images were acquired on a Zeiss 510 upright confocal microscope using a 40× objective.

### Immunocytochemistry and visualization of cell fills

The following reagents were used for immunostaining against tyrosine hydroxylase and visualization of neurobiotin (cell fills): mouse anti-tyrosine hydroxylase (Immunostar, Hudson, WI, 1:50), Alexa Fluor 633 goat anti-mouse (Life Technologies, 1:400), and Alexa Fluor 568 streptavidin (Life Technologies, 1:300). Brains were dissected, fixed for 20 min in 4% PFA, rinsed 3 × 10 min in PBST, and placed in primary antibodies in PBST at 4° for 1–3 days. Brains were next rinsed 3 × 10 min in PBST and placed in secondary antibodies and/or streptavidin in PBST at 4° for 1–3 days. Finally, brains were rinsed 3 × 10 min in PBST and mounted in Vectashield for confocal imaging.

### Whole-cell electrophysiological recordings

Flies were anesthetized on ice and placed into a custom-made chamber, the head was rotated 180 degrees, and the fly was secured in place using UV-curable glue. Three to four-day-old females were used because the glial sheath surrounding the brain in flies in this age range is easiest to remove to assess DA-DNs for electrophysiological recordings. Because DA-DNs are situated medially but lie anterior to the neck connective (and are hence not accessible from the posterior face of the brain), head rotation was necessary to access these cells for electrophysiological recordings. Legs were painted with a black marker (Copic black multiliner, 0.03 mm) or acrylic paints to enhance contrast and allow tracking of leg movements. A small opening was made in the cuticle overlying the posterior subesophageal zone (SEZ) after covering the head and dorsal half of the fly's body with external saline (103 mmol/L NaCl, 5 mmol/L KCl, 5 mmol/L Tris, 10 mmol/L glucose, 26 mmol/L NaHC0_3_, 1 mmol/L NaH_2_P04, 1.5 mmol/L CaCl_2_, 4 mmol/L MgCl_2_, osmolarity adjusted to 270–285 mOsm, bubbled with 95% O_2_/5% CO_2_ to pH 7.1–7.4). The trachea and perineuronal sheath were removed with fine forceps. A cluster of 3 TH-GAL4 neurons resides in the posterior SEZ, consisting of the left and right DA-DNs and the midline TH-VUM neuron (Marella et al. 2012). Left and right DA-DNs were targeted for recording by expression of GFP, visualized using an Olympus BX51W1 upright microscope equipped with epifluorescence and standard filters. Glass electrodes containing internal solution were used to carry out whole-cell recordings (electrode resistance ranged from 5 to 10 MΩ; 140 mmol/L K-aspartate, 1 mmol/L KCl, 10 mmol/L HEPES, 1 mmol/L EGTA, 0.5 mmol/L Na_3_GTP, 4 mmol/L MgATP, 265–270 mOsm, pH 7.1–7.4, often containing 1% neurobiotin, allowed to passively diffuse into the cell over the duration of each recording). Voltage was recorded at 10 kHz using a model 2400 patch-clamp amplifier (A-M systems) and low-pass filtered at 5 kHz.

The identity of the recorded DA-DN (left or right) was established using three criteria. First, because DA-DNs are located lateral and a bit posterior to the TH-VUM, the identification of the recorded cells based on anatomical orientation was usually unambiguous. Second, a small number of whole-cell recordings were performed from the TH-VUM (data not shown), and the physiology of this cell (both tonic activity and sensory responses) was distinct from the DA-DNs. Thus, it was obvious from the physiological data whether we were recording from a DA-DN or the TH-VUM. Third, although we did not obtain complete cell fills during every recording, we obtained partial cell fills in almost every recording, and the lateralization of the recorded cell's arborizations within the SEZ was used as an additional criterion to confirm the identity of the recorded DA-DN.

Spikes were identified by detecting peaks in the first derivative of the voltage trace using a custom Matlab script. Instantaneous firing rates (IFRs) were calculated for single trials for comparison with behavioral data by smoothing spike trains with a 200 msec moving average filter. Peristimulus time histograms (PSTHs) were created by averaging IFRs from multiple trials collected in the same stimulus conditions. DA-DN spikes were categorized as tonic or burst spikes by setting an inter-spike interval threshold for each cell (usually 150 msec). After detection of all spike times in a trial, tonic spikes were defined as those that were both preceded and followed by inter-spike intervals greater than the threshold. The first spike in a burst was defined as being preceded by an inter-spike interval greater than the threshold and followed by an inter-spike interval less that the threshold. To compare the timing of DA-DN burst spikes to the onset of leg movements, isolated leg movements were selected that were preceded by at least 0.5 sec of inactivity (i.e., total leg speed = 0). Analysis was restricted to isolated movements that were associated with DA-DN burst spikes.

Injections of positive or negative current during whole-cell recordings were used to manipulate spike rates in single DA-DNs (−100 to −300 pA to decrease spike rate and 100 to 300 pA to increase spike rate). To increase spike rates in both DA-DNs, TH-GAL4 flies were crossed to flies expressing P2X2, an exogenous cation channel activated by ATP (UAS-P2X2 fly line, Lima and Miesenböck 2005). To focally apply ATP to DA-DNs, a pipette was positioned in close proximity to the DA-DN cell bodies, and a small amount of positive pressure was used to deliver ATP (10 mmol/L) to the cells throughout the duration of the trial (∽15 sec total application, started ∽5 sec before start of 10 sec-long trial). Whole-cell recordings from DA-DNs were carried out in parallel with ATP application to verify effects on spike rate and to optimize ATP application timing and duration in a subset of the experiments (*N* = 3 recordings from UAS-GFP;TH-GAL4/UAS-P2X2 flies included in behavioral data set, *N* = 3 additional recordings from flies in which behavior was not measured but effects of ATP on spike rate were confirmed), and the remainder of the experiments were carried out with an identical protocol for ATP application but without whole-cell recordings (N = 4 additional experiments, behavior only).

### Acquisition of video data

A camera (Marlin F131B or F131C IRF, Allied Vision Technologies, Exton, PA) was placed below the fly to capture videos of leg movements. In experiments using color cameras, an external light source (ACE Fiber Optic, Schott, Southbridge, MA) with a white light filter was used to illuminate the fly and enhance the appearance of colors in the video; otherwise, the light source (TH4-100 power supply, Olympus, Center Valley, PA) on an Olympus BX51W1 upright microscope was filtered (IR bandpass) and used to illuminate the fly. Custom Matlab software was used to trigger acquisition of video frames (70–80 Hz) and acquisition of electrophysiological data at approximately the same time. In early experiments, we used video timestamps from Matlab's image acquisition toolbox to determine the timing of each video frame. We discovered that although these video timestamps were internally consistent (i.e., ∽125 msec interval between consecutive frames collected at 80 Hz), these timestamps were slightly misaligned to the timestamps of the electrophysiological data generated by Matlab's data acquisition toolbox. To implement a post hoc correction of video timestamps in these experiments, we determined the average error in alignment using light stimulus trials. A command pulse to open the shutter was sent in synchrony with a command pulse to trigger frame acquisition from the camera. The timestamp for these command pulses generated by the data acquisition toolbox was then compared to the timestamp of the video frame in which the light appeared. The average error was −81.4 ± 6.1 msec (calculated from 18 light trials), and video timestamps were corrected by this average value. In subsequent experiments, video timestamps were estimated as the time of the command pulses sent to trigger frame acquisition plus the exposure time of the camera. A comparison of the cross-covariance between spike rate and leg movements for DA-DNs in which video timestamps were calculated in these two ways revealed no difference in covariance coefficient or width (data not shown), indicating that our post hoc correction has not obscured a more precise relationship between DA-DN spikes and leg movements.

### Analysis of leg movements

Leg movements were manually scored as the change in position of each leg tip across pairs of consecutive video frames, and total leg speed was calculated as the sum of the movement of all six legs across pairs of frames. The distance of each leg tip from the body in each frame was estimated by measuring the distance between the leg tip and the coxa-trochanter joint (i.e., near the point of leg attachment to the body). Position and movement vectors calculated within/across video frames were linearly interpolated to the timestamps of the electrophysiological data in order to compare neural activity with behavioral data. To obtain a measure of leg movement speed that was independent of movement probability, mean speed was calculated for each trial by excluding time points of inactivity and then dividing total leg speed at the remaining time points by the inverse of the video frame rate (range was 70–80 frames/s). These values were averaged to obtain the mean movement speed for each trial. Similarly, we calculated the mean number of legs contributing to movement in each trial by excluding time points of inactivity so that the measure was not influenced by movement probability. The number of legs moving was counted at each remaining time point (values ranging from 1 to 6), and these values were averaged to obtain the mean number of legs contributing to movement for each trial.

### Comparison of DA-DN spike rates to behavior

The cross-covariance between DA-DN spike rates (IFRs for single trials) and leg speed/position was calculated for each trial using the xcov function in Matlab with the “coeff” scaling option, which normalizes each vector such that the auto-covariance at time lag 0 equals 1. Thus, each cross-covariance has a maximum possible value of 1 and a minimum possible value of −1, allowing us to pool data across cells and compare the strength of the cross-covariance across groups/conditions. Cross-covariances for multiple trials of the same type were then averaged together for each cell to obtain a trial-averaged cross-covariance. The trial-averaged covariance coefficient (C.C.) was calculated as the maximum value of the trial-averaged cross-covariance between time lags of −1 and 1 sec. The C.C. for a given trial was calculated by measuring the value of the single trial cross-covariance at the time lag of the trial-averaged C.C. To calculate the significance of the cross-covariance between spike rate and leg speed/position for individual DA-DNs, C.C.s were calculated for matched trials (i.e., trial 1 IFR vs. trial 1 movement, etc.) and for shuffled trial comparisons (i.e., trial 1 IFR vs. trial 2 movement, etc.). Significant differences between matched and shuffled C.C. distributions were detected using the Mann–Whitney *U*-test.

### Presentation of sensory stimuli

Delivery of mechanosensory, odor, and light stimuli was controlled via a pulse generator (505 Pulse Generator, Berkeley Nucleonics, San Rafael, CA) and custom Matlab software. Air was directed at the fly using plastic tubing and was controlled via two computer-controlled solenoid valves. For the mechanosensory stimulus, an air stream (2.2 L/min total flow rate) was directed at the fly for 2 sec. During odor trials, clean air (2.2 L/min total flow rate) was turned on 2 sec before presentation of a 1 sec-long odor stimulus and turned off 2 sec after the end of the odor stimulus. The odor stimulus was delivered using a solenoid valve to divert a small air stream (0.2 L/min) through a glass vial containing the odorant before rejoining the main air stream (2 L/min). This stimulus structure keeps the total air flow constant during the stimulus period and was designed to allow us to distinguish odor responses from mechanosensory responses. However, flies often move their legs at the onset of the air flow and prior to delivery of the odor stimulus, making it difficult to distinguish sensory responses to odors from movement-related activity. To address this issue, a subset of odor trials were carried out in which the clean air stream was turned on and directed at the fly prior to the beginning of each trial and was left on throughout the collection of odor trial data. Odorants used were pure apple cider vinegar, trans-2-hexenal (10^−2^ in paraffin oil), and ethyl butyrate (10^−2^ in paraffin oil). During light trials, an external light source (Schott ACE) was used to illuminate the fly and was covered with red filter paper (Roscolux #27, medium red), providing enough illumination for the cameras to record the fly's behavior but at a wavelength that flies cannot see well. A computer-controlled shutter was then used to deliver a 1 sec duration light stimulus to the fly using the microscope light source (Olympus TH4-100 power supply, set to 25%, 50%, or 100% maximum intensity).

## Results

### Identification and anatomical description of *Drosophila* DA-DNs

**Figure 1:**
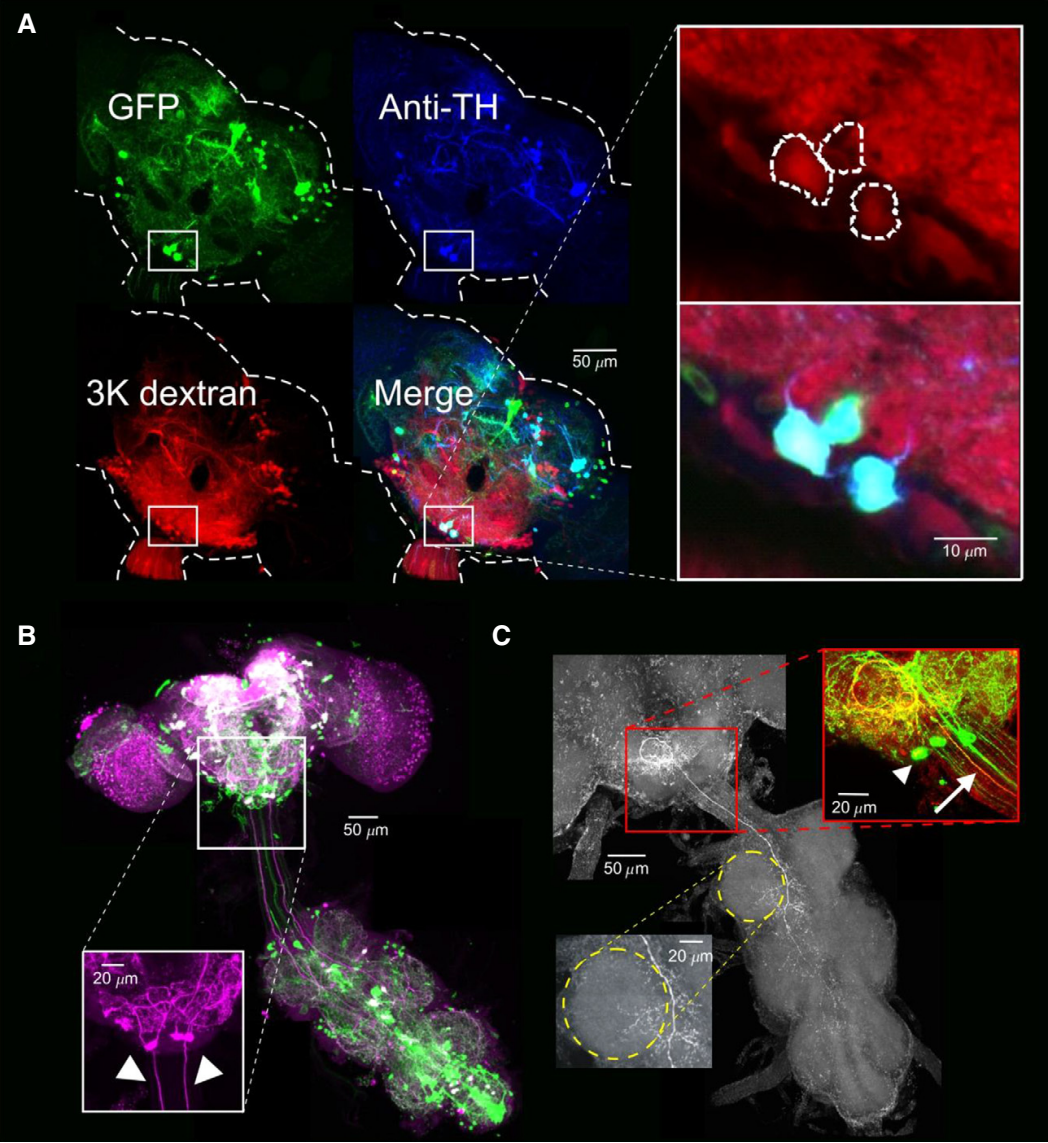
Bulk-labeling and anti-TH labeling reveal 2 DA-DNs in the *Drosophila* brain. (A) A UAS-mCD8::GFP;TH-GAL4 brain has two triple-labeled SEZ neurons (GFP, green; anti-TH, blue; Alexa 594 dextran, red; maximum projection image viewed from anterior side of brain, *z*-interval=3 *μ*m; note that projection only includes image sections from the posterior brain). Inset shows zoom of a single optical section from indicated region. (B) Anti-TH labeling (magenta) of a UAS-mCD8::GFP;TH-GAL4 brain shows that left and right DA-DNs send projections into the VNC. Arrowheads in inset mark DA-DN axons (maximum projection images viewed from anterior side of brain, ventral side of VNC, *z*-interval = 3 *μ*m). (C) Representative cell fill of a DA-DN obtained during whole-cell recording (red streptavidin; image converted to grayscale for presentation). Left inset shows innervation of ipsilateral leg motor neuropil in the VNC (yellow dotted circle). Right inset shows overlap between the cell fill (red) and GFP (green) in the indicated region within the SEZ. Arrowhead marks the soma of the filled cell. Arrow marks double-labeled DA-DN axon (maximum projection images viewed from anterior side of brain, ventral side of VNC, *z*-interval = 2 *μ*m; see also Supplemental Movies S1–S3.

To label putative DA-DNs, we retrogradely labeled DN cell bodies by applying dextran to the severed necks of UAS::mCD8GFP;TH-GAL4 flies (see Materials and Methods). In these flies, the yeast transcriptional activator GAL4 acts on the UAS enhancer to drive GFP expression in neurons that express the enzyme tyrosine hydroxylase (TH), which catalyzes the creation of a dopamine precursor (Fig.1A,B; ∽300 neurons labeled throughout the brain and ventral nerve cord (VNC); Mao and Davis 2009). Because the TH-GAL4 line is not a perfect reporter of brain TH expression (Friggi-Grelin et al. 2003; Mao and Davis 2009), brains were also reacted with a TH antibody whose specificity has been validated in a TH CNS null *Drosophila* mutant (Riemensperger et al. 2011). Notably, we found only two cell bodies that were TH-positive, GFP-positive, and bulk-labeled, a midline pair of neurons in the posterior subesophageal zone (SEZ) (Fig.1A; left and right DA-DNs; 2 TH-positive, GFP-positive, and bulk-labeled neurons were observed consistently in *N* = 8 brains). Interestingly, we observed that two other SEZ neurons were both GFP-positive and TH-positive, but these cells were never bulk-labeled and do not have descending axons (one neuron located in the same posterior SEZ cluster as DA-DNs (TH-VUM neuron, Marella et al. 2012) and one neuron in the anterior SEZ, data not shown). We note that the location and number of DA-DN cell bodies in *Drosophila* bears similarity to TH-positive and DA-immunoreactive neurons identified in the SEZ of the blowfly *Calliphora*, although all four SEZ DA neurons send descending projections to the VNC in the blowfly (Nässel and Elekes 1992). Our findings suggest that only two dopaminergic neurons provide descending input to body motor circuits in the *Drosophila* VNC, greatly simplifying the goal of characterizing the relationship of DA-DN activity to behavior.

We next characterized the morphology of the two DA-DNs. Labeling of the intact nervous system with the TH antibody revealed that DA-DNs send descending projections into the VNC via two large caliber axons (*N* = 6 brains, Fig.1B). We also used the TH-GAL4 driver to drive expression of a construct that labels axonal and dendritic arborizations in green and red, respectively, and confirmed that these descending projections are axonal (data not shown, *N* = 3 brains; UAS-DenMark, UAS-syt.eGFP, Nicolaï et al. 2010). To visualize the projections of DA-DNs within the brain and VNC, individual DA-DNs were filled with neurobiotin during whole-cell recordings (Fig.1C; see below for electrophysiological data from these and other DA-DNs). Our cell fills (*N* = 4) revealed that, within the brain, left and right DA-DNs have mirror symmetric projections that are largely restricted to the SEZ, with denser innervation ipsilateral to the cell body. DA-DNs also have arborizations that infringe upon the edges of the ipsilateral antennal mechanosensory and motor center (see Supplemental Movies S1–S3 for entire image stack of cell fill shown in Fig.1C). Within the VNC, the left and right DA-DNs innervate leg neuropil ipsilaterally and dorsally, where leg motor circuits are known to reside (Baek and Mann 2009; Brierley et al. 2012), with a smaller amount of innervation in the contralateral VNC, as well as innervation along the midline longitudinal tracts (Fig.1C; dotted yellow circle denotes ipsilateral foreleg neuropil; see also Supplemental Movies S1–S3). We note that the left and right DA-DNs appear to be the same cells as those whose projections within the brain have been described by Marella et al. 2012 (see Fig. S2 from Marella et al. 2012), although the projections of these cells to the VNC have not been previously described. Although DA-DNs may influence many types of motor output, these anatomical findings motivated us to focus on the relationship of DA-DN activity to leg movements.

### DA-DN spike rates increase during spontaneous leg movements

**Figure 2:**
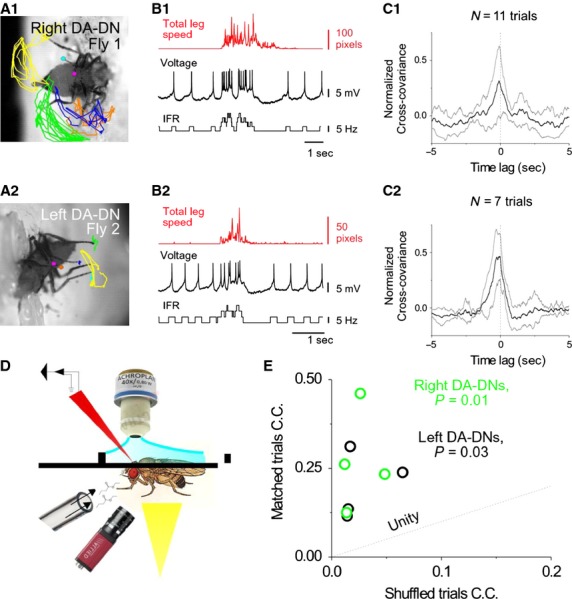
Spike rates in DA-DNs increase during leg movements. (A1–A2) Plots of leg positions of two flies during single trials with spontaneous leg movements (left foreleg, orange; right foreleg, magenta; left midleg, blue; right midleg, cyan; left hindleg, green; right hindleg, yellow. (B1–B2) Top: Total leg speed (sum of change in position of all 6 legs measured across each pair of frames) is plotted for the trials shown in (A1–A2). Middle: Voltage traces obtained from whole-cell recordings from a right DA-DN (B1) and a left DA-DN (B2) during these same trials. Bottom: Instantaneous firing rate (IFRs) for these trials. (C1–C2) Mean normalized cross-covariance between spike rate and total leg speed is plotted for spontaneous trials for the flies/cells in (A–B) (gray lines, ±1 SD). (D) Schematic of whole-cell recording with simultaneous monitoring of behavior and presentation of sensory stimuli. (E) For each DA-DN, the covariance coefficient (C.C.) was calculated for the cross-covariance between spike rate and total leg speed for matched and for shuffled spontaneous trials. Left DA-DNs, black, *P* = 0.03; Right DA-DNs, green, *P* = 0.01.

To determine whether DA-DN activity is related to leg movements, we developed a preparation that allowed us to perform whole-cell recordings from DA-DNs and to simultaneously observe spontaneous and stimulus-evoked leg movements. Flies were secured in a custom-made recording chamber, legs were colored with markers or paint to aid in the tracking of leg tips, and movement was measured for each leg as the change in leg tip position between consecutive video frames (frames rates were 70–80 Hz, Fig.2A,D; see Materials and Methods). Flies exhibit robust leg movements in our preparation (15/17 flies exhibited either spontaneous or stimulus-evoked leg movements). We first assessed the relationship between DA-DN activity and leg movements in spontaneous trials (no sensory stimuli presented). In these initial comparisons, total leg speed was calculated by adding together the total change in position for each of the six legs across consecutive video frames (i.e., total leg speed is total leg movement over a fixed time interval; position information and leg identity were discarded for the time being). During time periods without leg movements, DA-DNs exhibited regular tonic firing (Fig.2B; mean spontaneous spike rate was 3.1 ± 1.9 Hz, *N* = 10 neurons). This pattern of activity changed dramatically during periods of leg movements. We observed that DA-DNs frequently switched from tonic to burst firing around the time that leg movements began and returned to tonic firing when movements ended (changes from tonic to burst firing observed in 36/65 trials with leg movements, data from *N* = 8 flies/DA-DNs; Fig.2B; total leg speed plotted in red; DA-DN voltage and instantaneous firing rate (IFR) plotted in black, see Materials and Methods for categorization of tonic and burst spikes). Although DA-DNs did not exhibit burst firing in all trials with leg movements, when burst spikes did occur, they were very reliably associated with leg movements (37 trials with DA-DN bursts, 36/37 had leg movements).

To quantify the relationship between spike rate and leg movements, we calculated the mean cross-covariance between spike rate (i.e., IFR) and total leg speed during spontaneous trials for individual left and right DA-DNs (Fig.2C). Most DA-DNs had spike rates that were positively and significantly correlated with total leg speed (*P* < 0.05 for 7/8 cells; significance tested by comparing covariance coefficients (C.C.) from matched and shuffled trial comparisons, Mann–Whitney *U*-test; see Materials and Methods). Whether considered as separate groups or as a single group, spike rates in both left and right DA-DNs were significantly correlated with total leg speed (Fig.2E; *P* < 0.01 for left DA-DNs, *P* < 0.03 for right DA-DNs, *P* < 0.001 for all DA-DNs as a single group; mean matched trials C.C. vs. mean shuffled trials C.C., paired *t*-test). These findings support the idea that DA-DN spike rates are significantly and positively correlated with spontaneous leg movements.

### Increases in DA-DN spike rate follow movement onset but precede increases in total leg speed

After finding that DA-DN spike rates increase during some leg movements, we next investigated whether increased spike rates were associated specifically with movement initiation. Although DA-DN bursting activity occurs approximately throughout the duration of the movement (Fig.2B), it is possible that the first spikes in these bursts are related to movement initiation. In this case, one would expect that the first DA-DN burst spike should precede movement onset.

**Figure 3:**
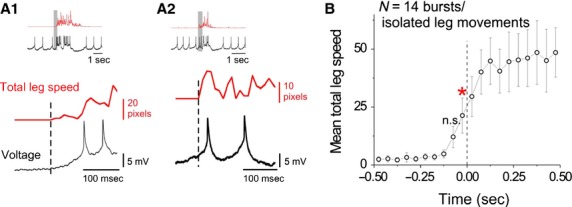
Increases in DA-DN spike rate follow movement onset. (A1–A2) Total leg speed (red) and DA-DN voltage (black) are plotted for the same trials shown in Fig.2B1–B2. The same data shown in Fig.2 are re-plotted in the top row here, with the time range indicated by the gray shading shown on an expanded time scale below. (B) For isolated leg movements, the onset of movement is aligned to the timing of the first DA-DN burst spike that accompanies the movement. This analysis reveals that DA-DN burst spikes, on average, follow movement onset (*N* = 14 DA-DN bursts/isolated leg movements; red asterisk indicates first prespike time bin in which total leg speed is significantly higher than baseline; *P* < 0.01, Tukey's HSD performed after one-way ANOVA with repeated measures).

An examination of the cross-covariance between DA-DN spike rate and total leg speed suggests that, on average, increases in DA-DN spike rate precede increases in total leg speed (Fig.2C; mean time lag of C.C. = −74 ± 29 msec for *N* = 8 DA-DNs). However, the cross-covariance analysis describes the average timing relationship between spike rate and total leg speed across entire trials and does not allow us to selectively examine periods of movement initiation. To specifically examine the relationship of DA-DN spike rates to movement onsets, we restricted our analysis to time periods with isolated leg movements (i.e., preceded by 0.5 sec of inactivity) that were associated with bursts of spikes in DA-DNs and compared the timing of movement onset to the timing of the first DA-DN burst spike. These comparisons revealed that, on average, movement onset occurs slightly before (∽50 msec) the first DA-DN burst spike (Fig.3; total leg speed is significantly greater than baseline beginning in the bin from 0 to 50 msec before DA-DN burst spike, red asterisk, Tukey's HSD performed after one-way ANOVA with repeated measures; *N* = 14 isolated leg movements/DA-DN bursts from eight flies/DA-DNs). These findings are inconsistent with the idea that DA-DN activity is important for movement initiation, and we next examined how DA-DN activity relates to other aspects of leg movement.

### Increases in DA-DN spike rate are correlated with movements of all legs

**Figure 4:**
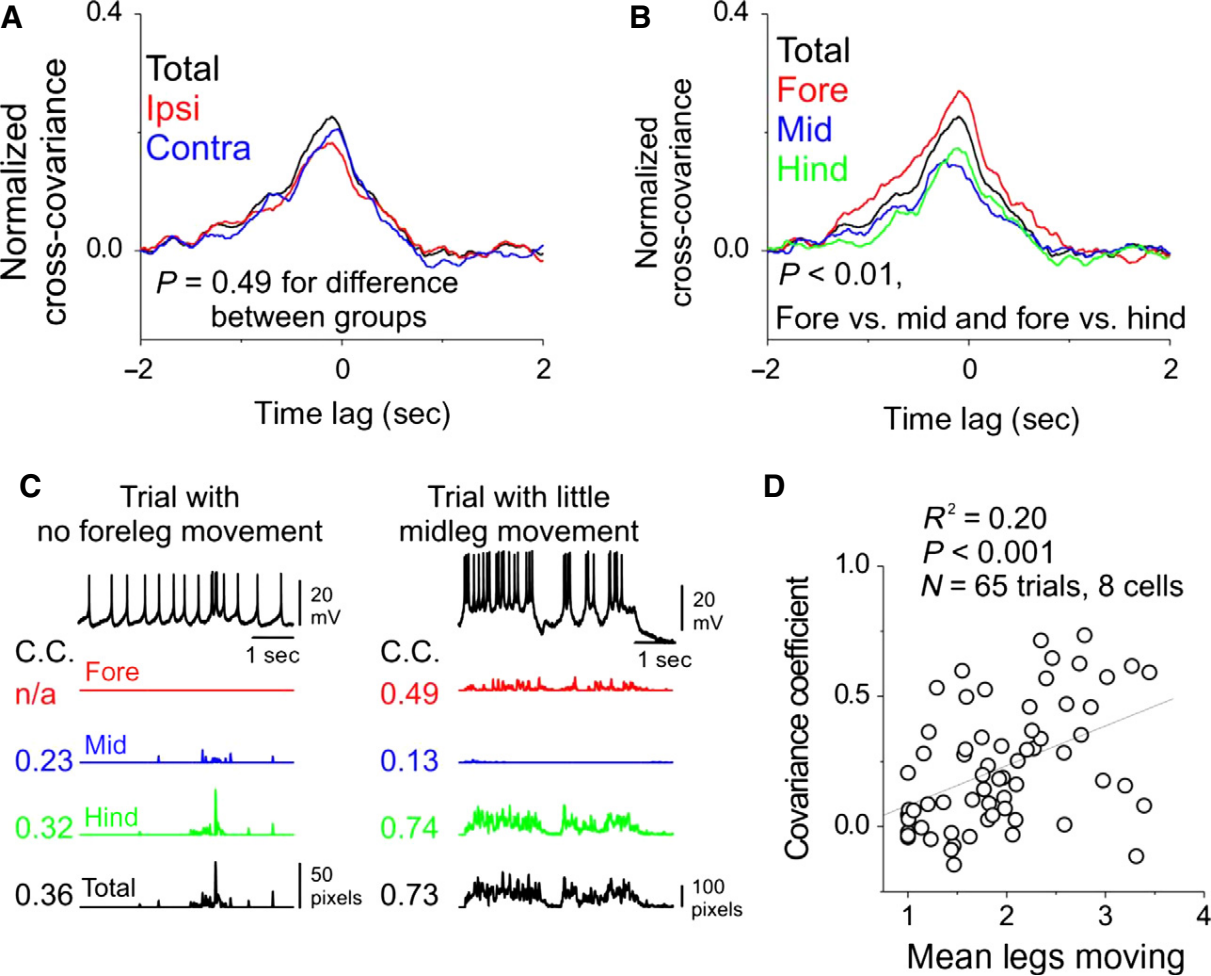
Spike rates in DA-DNs are related to movements of all legs. (A) Spike rates in DA-DNs are not significantly better related to the total speed of legs ipsilateral to their cell bodies (red) than they are to the total speed of contralateral legs (blue) or to the summed speed of all six legs (total leg speed, black). (B) DA-DN spike rates are significantly better related to total speed of the forelegs than to the midlegs or hindlegs (*P* < 0.01 for forelegs vs. midlegs and forelegs vs. hindlegs; Tukey's HSD performed after one-way ANOVA with repeated measures; *P* > 0.05 for difference between total leg speed and other leg sets). (C) Left: example trial without foreleg movements. Numbers indicate C.C. of the cross-covariance between spike rate and total speed of pairs of legs. Right: same, but for a trial in which the midlegs contribute little to total leg speed. (D) The mean number of legs contributing to movement in each trial is plotted against the C.C. of the cross-covariance between DA-DN spike rate and total leg speed (*R*^2^ = 0.20, *P* < 0.001).

Given that DA-DNs have denser projections to the side of the brain and the VNC ipsilateral to their cell bodies, we wondered whether spike rates in these cells were preferentially related to the movements of ipsilateral legs. To examine this idea, we calculated the cross-covariance between DA-DN spike rate and the summed speed of all legs (i.e., total leg speed), ipsilateral legs, or contralateral legs (Fig.4A). Interestingly, the peak cross-covariance was not significantly different for these three comparisons, suggesting that DA-DN spike rate is equally well related to speed summed over legs from either side of the body (mean C.C. for spike rate vs. total leg speed was 0.24 ± 0.11; vs. ipsi, 0.21 ± 0.13; vs. contra, 0.23 ± 0.10; *P* = 0.49 for difference between groups, *N* = 8 cells, one-way ANOVA with repeated measures). We also examined whether DA-DN activity was preferentially related to total speed of foreleg, midleg, or hindleg movements. DA-DN spike rates were not significantly better related to speed in any of these leg pairs than to total leg speed (Fig.4B; mean C.C. for spike rate vs. total leg speed was 0.24 ± 0.11; vs. forelegs, 0.30 ± 0.12; vs. midlegs, 0.18 ± 0.08; vs. hindlegs, 0.19 ± 0.13; *N* = 8 cells, *P* > 0.05, one-way ANOVA with repeated measures). However, foreleg movements were better related to DA-DN spike rate than were midleg or hindleg movements, perhaps reflecting the more pronounced projections of DA-DNs to the foreleg motor neuropil (Fig.1C and Supplemental Movie S2; *P* < 0.01 for forelegs vs. midlegs C.C.; *P* < 0.01 for forelegs vs. hindlegs C.C.; Tukey's HSD test performed after one-way ANOVA with repeated measures, N = 8 cells). Taken together, these findings indicate that DA-DN spike rate is significantly related to the movement of all legs, with a small bias toward the forelegs.

Two observations further support the conclusion that DA-DN spike rate is related to the movements of all legs. First, example trials can be found in which a given set of legs doesn't move or moves very little and yet DA-DN spike rate is still significantly correlated with total speed of the other legs (Fig.4C; examples shown for trials with little or no foreleg or midleg movement; movements without contribution from the hindlegs were extremely rare). Second, we reasoned that if DA-DNs are recruited during movements of any subset of the 6 legs, DA-DN spike rates may be better correlated with movements that included a larger number of legs. Indeed, although flies in our preparation rarely move a single leg in isolation, when this type of movement was observed, we never saw a corresponding increase in DA-DN spike rate (data not shown). To test this idea more rigorously, we compared the mean number of legs moving in each trial to the covariance coefficient for the comparison of DA-DN spike rate and total leg speed for that same trial (Fig.4D; mean number of legs moving was counted at each time point and averaged for each trial after excluding time points of inactivity). This analysis revealed that DA-DN spike rates were significantly better related to leg movements when more legs contributed to the movement (*R*^2^ = 0.20 for linear regression, *P* < 0.001, *N* = 65 spontaneous trials from 8 flies/cells). In summary, these observations support the idea that DA-DN activity is related to the movements of all of the legs, rather than to a specific subset.

### DA-DNs are differentially recruited during different behavioral categories of leg movements

Although DA-DN spike rates were significantly correlated with total leg speed, this relationship was stronger in some spontaneous trials than in others (i.e., larger C.C. for comparison of DA-DN spike rate and total leg speed; see below and Fig.4D). This observation, in addition to our earlier observation that DA-DN burst spikes were only associated with a subset of trials that contained leg movements (36/65 trials) raises the idea that DA-DN burst activity may be recruited preferentially during certain types of leg movements. To begin addressing this question, we tested whether DA-DN activity was better related to certain behavioral categories of leg movement.

**Figure 5:**
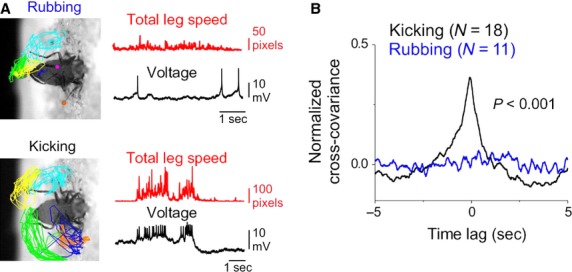
Spike rates in left and right DA-DNs are better related to kicking than to rubbing leg movements. (A) Leg position plots (left), total leg speed (red), and voltage traces from DA-DN whole-cell recordings (black) are shown for a rubbing and a kicking trial from the same fly (left foreleg, orange; right foreleg, magenta; left midleg, blue; right midleg, cyan; left hindleg, green; right hindleg, yellow). (B) Mean cross-covariance of DA-DN spike rate and total leg speed is plotted for kicking trials (black; *N* = 18 trials, five flies) and rubbing trials (blue; *N* = 11 trials, five flies). DA-DN spike rate is significantly better related to kicking than to rubbing (*P* < 0.001 for difference in C.C., *t*-test).

In our preparation, there is a great diversity of movement in terms of the number and identity of legs involved and the speed of movement, making it difficult to classify every aspect of an ongoing leg movement into a discrete behavioral category. However, two types of leg movements were observed frequently across many flies. First, all flies that exhibited leg movements in the electrophysiological preparation engaged in “kicking” leg movements that are characterized by rapid arc-like movements of the hindlegs (15/15 flies; Fig.5A and Supplemental Movie S4). Second, a subset of these flies (5/15) also engaged in “rubbing” movements of the hindlegs and midlegs that are reminiscent of grooming leg movements (Fig.5A and Supplemental Movie S5). The categorical labels “kicking” and “rubbing” are therefore used to describe two frequently observed types of leg movement but are not meant to equate them with behaviors in unrestrained flies (Szebenyi 1969; Connolly and Cook 1973; Phillis et al. 1993; Seeds et al. 2014). Kicking and rubbing leg movements were readily identified and distinguished from one another based on the positions occupied by the hindlegs (Fig.5A) and the speed of leg movements (see below). When we compared the relationship between DA-DN spike rate and total leg speed in trials that included either kicking or rubbing but not both types of leg movements, we noted that DA-DN spike rates were well related to kicking and poorly related to rubbing movements (Fig.5A,B; *N* = 18 kicking trials, *N* = 11 rubbing trials from five flies; mean C.C. for kicking trials was 0.37 ± 0.06; mean C.C. for rubbing trials was 0.01 ± 0.02). These findings support the idea that DA-DNs are preferentially recruited during different categories of leg movements.

What differences between kicking and rubbing might explain the differential recruitment of DA-DNs? One possibility is that differences in the features of leg movements (i.e., speed, legs involved, etc.) account for this observation. Indeed, leg movements are faster in kicking trials than in rubbing trials (kicking mean speed = 1840 ± 333 pixels/s, rubbing mean speed = 841 ± 121 pixels/s; mean speed is the trial-averaged value of total leg speed divided by frame rate, calculated after excluding time points of inactivity), and a comparison of the mean speed in each kicking trial to the peak cross-covariance between spike rate and total leg speed for that trial revealed a significant relationship (data not shown, *R*^2^ = 0.19 for linear regression, *P* = 0.02; *N* = 27 trials, eight flies/cells). However, when we considered only kicking trials that fall within the same mean speed range as rubbing trials (*N* = 10 low speed kicking trials; mean speed < 1700 pixels/s), we found that DA-DN spike rates are still significantly better correlated with leg movements in low speed kicking trials than in rubbing trials (low speed kicking, mean speed = 896 ± 139 pixels/s, mean C.C. = 0.24 ± 0.08; rubbing mean speed = 841 ± 121 pixels/s, mean C.C. = 0.01 ± 0.02; *P* < 0.01 for difference in C.C.). We also found no significant difference in the total number of legs involved in kicking and rubbing trials (data not shown), indicating that this movement feature cannot explain the differential relationship of DA-DN activity to these two behavior types. Taken together, these data do not support the idea that differences in leg movement features account for the differential recruitment of DA-DNs during kicking and rubbing. Instead, it is likely that DA-DNs participate only in a subset of behavioral categories of leg movements.

### DA-DN responses to sensory stimuli

**Figure 6:**
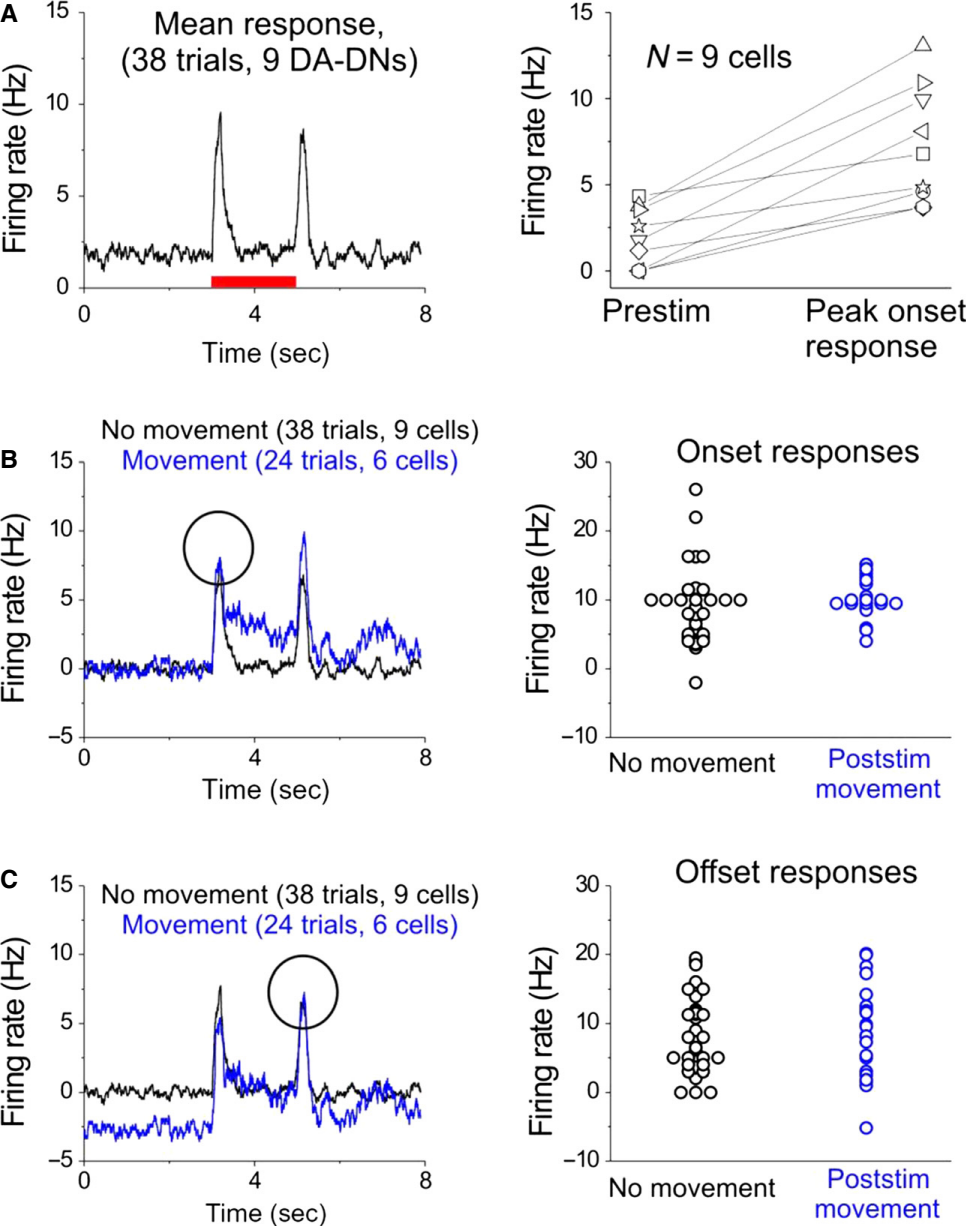
DA-DN mechanosensory responses do not predict subsequent movement. (A) Left: PSTH shows mean response to mechanosensory stimulus in trials without leg movement. Right: Mean change in firing rate shown for each cell after stimulus onset. (B–C) Mean DA-DN firing rates are compared during mechanosensory trials with (blue) and without (black) leg movement after stimulus presentation. (B) Prestimulus firing rate is subtracted from each trace to examine onset responses to stimulus. Left: mean PSTHs; Right: Onset responses for all cells. (C) Poststimulus firing rate is subtracted from each trace to examine offset responses. All panels as in (B).

Many descending neurons mediate specific sensorimotor transformations, whereby a specific sensory stimulus elicits spikes, and these spikes precede and predict subsequent initiation, modulation, or inhibition of behavior (Tanouye and Wyman 1980; Möhl and Bacon 1983; Hedwig 2000; Perrins et al. 2002; Li et al. 2003; Fotowat et al. 2011; Kohatsu et al. 2011; Severi et al. 2014). To evaluate whether DA-DNs might play a role in mediating behavioral responses to specific sensory stimuli, we next measured DA-DN responses to sensory stimuli and investigated the relationship of DA-DN activity to stimulus-induced leg movements. Flies were presented with a small panel of attractive odors (apple cider vinegar, trans-2-hexenal, and ethyl butyrate), a mechanosensory stimulus (air puff applied to the head and body), and a visual stimulus (1 sec duration light pulse). Although these stimuli often elicit behavioral responses (see below), we first assessed DA-DN responses to sensory stimuli in trials without leg movements. Both left and right DA-DNs exhibit small onset and offset responses to the mechanosensory stimulus (Fig.6A), usually consisting of a burst of 2–3 spikes. In contrast, these cells do not exhibit detectable responses to any of the three odors that we tested (data not shown). Finally, some DA-DNs responded to a light stimulus, although these responses were variable and were only observed at extremely high (and likely aversive) light intensities (data not shown). In summary, DA-DNs respond relatively selectively to a mechanosensory stimulus, and we next examined the relationship of these sensory responses to leg movement-related DA-DN activity and behavior.

### DA-DN sensory responses do not predict subsequent movement

To test whether DA-DN responses to the mechanosensory stimulus were predictive of subsequent movement, we compared DA-DN activity in trials in which stimulus presentation elicited movement to those in which it did not (Fig.6B,C). Notably, the peak onset response to the mechanosensory stimulus was not different between trials with and without movement (Fig.6B), nor did the latency of the first poststimulus spike differ, indicating that the strength and latency of mechanosensory responses in DA-DNs do not predict subsequent movement (mean latency, 110 ± 12 msec for trial with movements, 144 ± 13 msec for trials without movement, *P* = 0.4, Mann–Whitney *U*-test; latency includes the time from computer-controlled opening of the solenoid valve till the air stream reaches the fly). Furthermore, in mechanosensory trials with movement, the latency of the first poststimulus spike was more tightly linked to stimulus onset than to movement onset, supporting the idea that these sensory responses are independent of subsequent movement (mean latency from stimulus onset to first poststimulus spike, 110 ± 12 msec; mean latency from first poststimulus spike to first poststimulus leg movement, 1048 ± 230 msec).

Following the onset response to the mechanosensory stimulus, a sustained increase in DA-DN spike rate can be seen in trials with movement (i.e., movement-related activity), followed by a time-locked increase in spike rate that occurs after the offset of the mechanosensory stimulus (Fig.6B, left panel, blue trace). Thus, movement-related activity does not eliminate or mask mechanosensory responses in DA-DNs. To examine the interaction of sensory- and movement-related activity in DA-DNs, we subtracted the mean movement-related increase in DA-DN firing rate from trials with movement and then compared the magnitude of the offset response between trials with and without movement. Notably, we found that offset responses are not different in trials with and without movement, indicating that mechanosensory and movement-related activity add linearly in DA-DNs (Fig.6C). These findings suggest that DA-DNs may receive independent inputs related to mechanosensory information and leg movement.

### Sensory context does not alter the relationship between DA-DN spike rate and leg movements

Although DA-DNs do not exhibit responses to many of the sensory stimuli we tested, these stimuli often elicit leg movements similar to those observed in spontaneous trials (data not shown). We used these trials to investigate whether the sensory context in which leg movements occur (spontaneous or in response to a stimulus) influences the relationship between DA-DN activity and total leg speed. We focused on trials in which pure apple cider vinegar was presented to the fly, because flies reliably moved their legs in response to this odor. The cross-covariance between DA-DN spike rate and total leg speed was calculated in these odor trials, revealing a significant relationship (data not shown; mean C.C. for odor trials was 0.27 ± 0.05; *P* < 0.05 for 8/8 DA-DNs; mean matched trials C.C. vs. mean shuffled trials C.C., paired *t*-test). However, a comparison to the cross-covariance of DA-DN spike rate and total leg speed in spontaneous trials revealed a similar strength of relationship, suggesting that stimulus presentation does not alter the relationship between DA-DN spike rate and behavior (*P* = 0.55 for difference in C.C. between spontaneous and odor trials, paired *t*-test, *N* = 8 cells). These findings suggest that DA-DNs exhibit similar movement-related activity, regardless of the sensory context in which leg movements are evoked.

### Increases in DA-DN firing rate do not elicit leg movements

**Figure 7:**
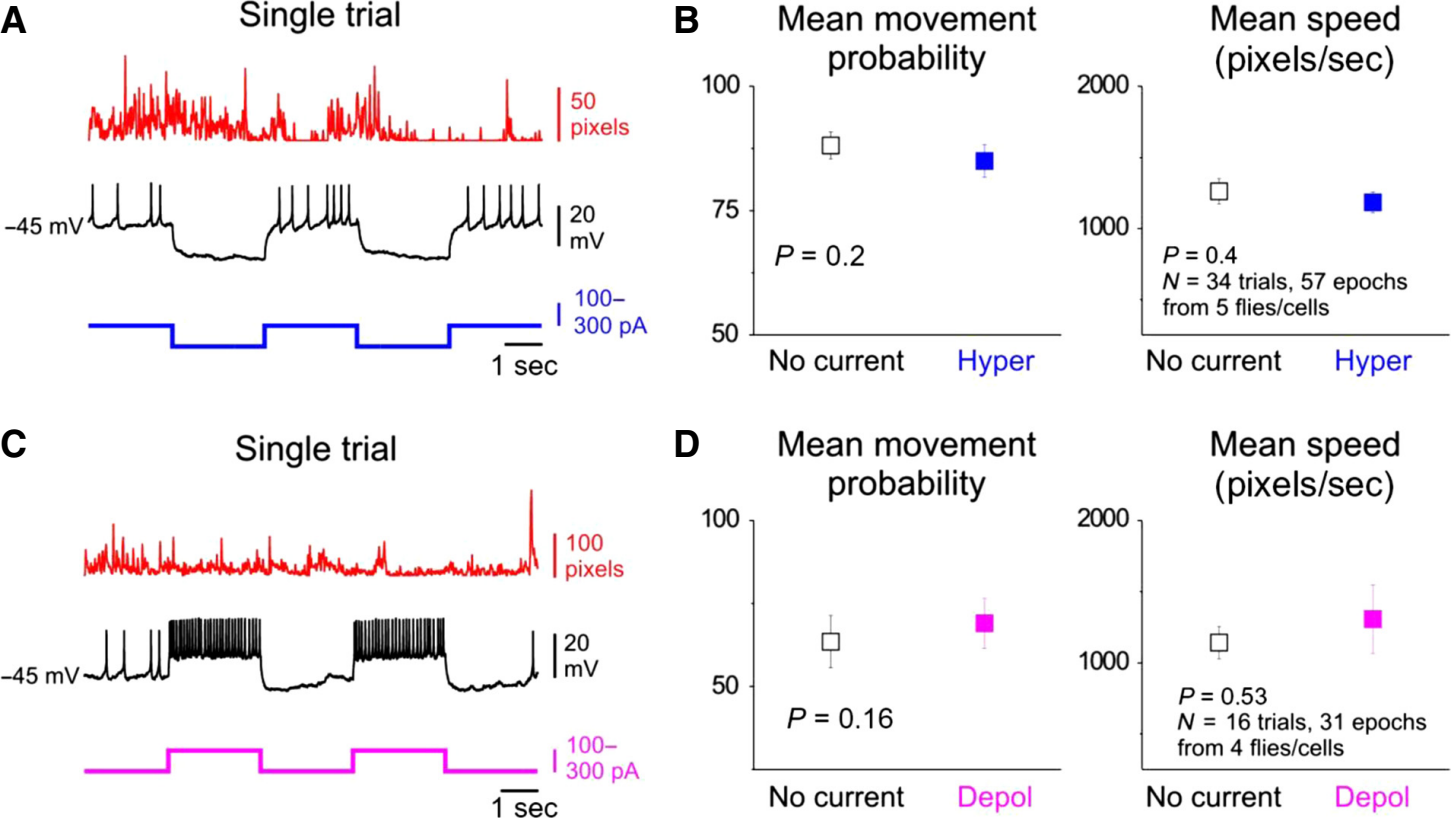
Bidirectional, acute manipulations of spike rate in single DA-DNs do not affect the probability or speed of ongoing leg movements. (A) Total leg speed (red) and voltage (black) are plotted for a trial in which hyperpolarizing current is injected during alternating epochs to decrease DA-DN spike rate. (B) Summary of mean movement probability and mean speed compared in epochs with and without current injection for all five flies/cells in the data set (*N* = 34 trials, 57 epochs, *P* > 0.05 for both comparisons). (C–D) All panels as in (A–B), showing experiments in which DA-DNs were depolarized to increase spike rate (*N* = 16 trials, 31 epochs from four flies/cells).

The observed correlation between leg movements and DA-DN spike rate raises the idea that DA-DN activity may influence leg movements. Based on reports that dopamine receptor agonists can elicit fictive locomotor rhythms in reduced body motor circuit preparations and even kicking and grooming in decapitated fruit flies (Claassen and Kammer 1986; Yellman et al. 1997; Tsyganov and Sakharov 2000; Puhl and Mesce 2008), we first tested whether activation of DA-DNs could elicit leg movements in intact flies by injecting depolarizing current into individual DA-DNs during whole-cell recordings (data not shown; 100 to 300 pA injected for 1.5–10 sec; *N* = 8 trials from three cells/flies). However, increasing DA-DN spike rates did not elicit movements during periods of inactivity, despite the fact that depolarization of DA-DNs drives spike rates in the same range that is observed during periods of leg movement (maximum spike rates elicited by depolarization ranged from 15 to 45 Hz; example of effects of depolarization on DA-DN spike rate shown in Fig.7C).

**Figure 8:**
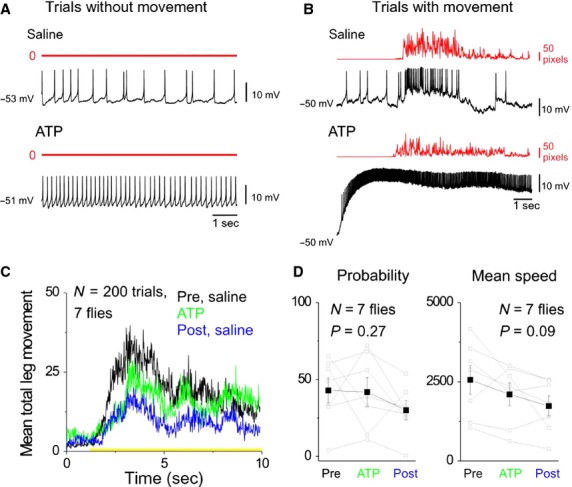
Increasing spike rate in both DA-DNs does not affect the probability or speed of leg movements. (A) Bottom two panels show an example trial in which ATP application to both DA-DNs increases spike rate in the recorded DA-DN but does not elicit leg movements (total leg speed = 0, red). For comparison, top two panels show a trial without leg movements from the same DA-DN recorded prior to ATP application. (B) Same as (A), but showing example saline and ATP trials with leg movement. (C) Plots of mean total leg speed for preATP trials (black), ATP trials (green), and post-ATP trials (blue) show that the behavioral response to odor presentation tends to decrease over time. Error bars omitted for clarity. Yellow bar indicates timing of odor stimulus. (D) Mean leg movement probability (left) and mean speed (right) are compared across preATP, ATP, and postATP trials for all flies in the data set (*N* = 7 flies; *P* > 0.05 for change in both parameters).

A remaining possibility is that left and right DA-DNs play complimentary roles in promoting movement and simultaneous activation of both cells is required to elicit movement in intact flies. To test this possibility, we used the TH-GAL4 driver to express a purinergic receptor, P2X2, in dopaminergic neurons (UAS-P2X2 flies from Lima and Miesenböck 2005). Fruit flies do not possess endogenous purinergic receptors, so only cells expressing TH-GAL4 will be activated by application by ATP. To spatially restrict the effects of the manipulation, we focally applied ATP to the posterior SEZ cluster of three TH-GAL4 neurons, which includes both DA-DNs (see Materials and Methods). In flies expressing the P2X2 receptor, we verified that ATP application rapidly and reversibly increased spike rates in DA-DNs (*N* = 6 flies/DA-DNs; ATP application started ∽5 sec prior to trial onset in Fig.8A; see also Fig.8B in which ATP application was started at trial onset). However, ATP application was not sufficient to elicit leg movements (example shown in Fig.8A; *N* = 21 trials from four flies), indicating that increased DA-DN activity is not sufficient to elicit leg movements in intact flies.

### Bidirectional manipulations of DA-DN activity do not affect leg movement probability or speed

Because DA-DN spike rates are rapidly modulated during certain categories of leg movements, we next investigated whether DA-DN activity modulates ongoing leg movements. In a first set of experiments, activity in single DA-DNs was acutely increased or decreased using current injections during whole-cell recordings (Fig.7). In some trials, a single current injection was delivered to the cell (duration of 2–2.5 sec), and in other trials, multiple alternating epochs of current injection were delivered (examples shown in Fig.7A,C). Leg movements during current injection were compared with those occurring in an equivalent length of time immediately preceding current injection. We first examined trials in which hyperpolarizing current was injected to decrease DA-DN activity (Fig.7A,B, -100–300 pA, *N* = 34 trials from five flies/cells, 57 epochs of hyperpolarization and 57 epochs of no current). Although hyperpolarization almost always completely eliminated DA-DN spikes (Fig.7A), we observed no obvious effect of decreasing DA-DN spike rates on total leg speed in single trials (Fig.7A; example trial shown for 1 fly/cell). We then compared movement probability and mean speed of leg movements from epochs with and without current injection for all flies in the data set and found no effect of hyperpolarization (Fig.7B; *N* = 5 flies/cells, *P* > 0.05 for both comparisons). We also verified that decreasing DA-DN spike rate had no effect on the movements of individual legs by comparing the speed distributions for each leg from epochs with and without current injection (data not shown; *P* > 0.05 for all legs, Mann–Whitney *U*-test). Finally, a comparison of position distributions for each leg revealed no effect of decreased DA-DN activity (data not shown; leg position quantified as distance from the body; *P* > 0.05 for all legs, Mann–Whitney *U*-test). We conclude that acutely decreasing activity in DA-DNs does not influence the probability or speed of ongoing leg movements in intact flies.

In a second set of experiments, we injected depolarizing current to transiently increase spike rates in single DA-DNs during whole-cell recordings (Fig.7C,D, 100–300 pA injected for 2–2.5 sec, *N* = 16 trials from four flies/cells, 31 epochs of depolarization and 31 epochs of no current). Although depolarization efficaciously increased DA-DN spike rates, an examination of individual trials revealed no effect of current injection on total leg speed (Fig.7C; example trial shown for 1 fly/cell). We also found that increasing DA-DN spike rate had no significant effect on movement probability or on the mean speed of leg movements across all flies in the data set (Fig.7D; *N* = 4 flies/cells, *P* > 0.05 for both comparisons) or the speed distributions of individual legs (data not shown; *P* > 0.05 for all legs, Mann–Whitney *U*-test). Finally, we saw no effect of current injection on leg positions (data not shown; *P* > 0.05 for all legs, Mann–Whitney *U*-test). In summary, we find that acute, bidirectional manipulations of spike rate in single DA-DNs do not influence the probability or speed of ongoing leg movements in intact flies.

A remaining possibility is that simultaneous manipulation of activity in both DA-DNs is required to drive a detectable influence on ongoing leg movements. To test this possibility, we again used the TH-GAL4 driver to express P2X2 in dopaminergic neurons, and ATP was focally applied to DA-DN cell bodies (see Materials and Methods). The robust effects of ATP application on DA-DN spike rate were confirmed with whole-cell recordings in three flies in which behavior was not measured and in three flies in which leg movements were tracked (see example in Fig.8B). In four additional flies, ATP application was performed in an identical manner while tracking leg movements but without an accompanying DA-DN whole-cell recording (*N* = 7 flies total with ATP application and leg tracking). The experiments were performed in blocks (9.7 ± 1.1 pre-ATP saline trials, followed by 10.0 ± 1.1 ATP trials, followed by 8.9 ± 1.5 post-ATP saline trials), and odor presentation (apple cider vinegar) was used to elicit leg movements in each trial.

In flies in which DA-DN whole-cell recordings were performed, an examination of ATP trials showed that drug application had a robust effect on DA-DN spike rate but no dramatic effect on the behavioral response to odor presentation as compared to pre-ATP trials (Fig.8B). To examine the effects of ATP application on leg movements across the seven flies in the data set, we compared the mean total leg speed that occurred in pre-ATP trials, ATP trials, and post-ATP trials (Fig.8C; error bars omitted for clarity). This comparison revealed a tendency for behavioral responses to apple cider vinegar to decrease across these three trial types, which was caused by a trend toward decreases in both movement probability and mean speed (Fig.8D; *P* > 0.05 for change in probability and speed across the seven flies). However, this trend toward decreased behavioral responsiveness was not different from that observed in control flies whose leg movements were tracked across 30 consecutive odor presentation trials (data not shown; *N* = 4 flies) and is likely attributable to the passage of time and/or behavioral habituation to the odor stimulus. Thus, ATP application and the accompanying depolarization of DA-DNs had no significant effect on leg movements. In addition, because drug application was prolonged (started ∽5 sec prior to trial plus 10 sec during each trial) and was repeated in multiple consecutive trials (10 consecutive ATP trials on average, each trial lasts 10 sec with an intertrial interval of ∽40 sec), our findings also suggest that repeated depolarization of DA-DNs does not have a cumulative effect on subsequent leg movements, at least over a time course of a few to 10 min. Thus, our observations are inconsistent with the idea that manipulations of DA-DN activity alone are sufficient to influence leg movements over a time scale of seconds to minutes in intact, behaving animals.

## Discussion

Descending dopaminergic projections to body motor circuits are present in a wide range of species (Commissiong and Sedgewick 1975; Björklund and Skagerberg 1979; Hökfelt et al. 1979; Nässel and Elekes 1992; McLean and Fetcho 2004), and we have for the first time identified DA-DNs in *Drosophila* and described their projections within both the brain (see also Nässel and Elekes 1992; Marella et al. 2012) and the VNC. To our knowledge, this is the first study to record the electrophysiological activity of identified DA-DNs in an intact and behaving animal, allowing us to address the long-standing issue of how DA-DN activity is related to movement in vivo.

### Relationship between DA-DN activity and leg movements

Studies in reduced body motor circuit preparations and spinalized animals report that bath application of or systemic treatment with dopamine receptor agonists elicits locomotor-like patterns of activity and/or limb movements, suggesting that DA-DNs may play an important role in promoting movement (Claassen and Kammer 1986; Yellman et al. 1997; Tsyganov and Sakharov 2000; Puhl and Mesce 2008; Lapointe et al. 2009). Although a major strength of these studies is the demonstration of dopamine's capacity to modulate the output of body motor circuits, they cannot reveal how and over what time course neural activity in dopaminergic inputs to body motor circuits relates to movement in intact animals. Using whole-cell recordings in behaving flies, we found that DA-DN spike rates are rapidly modulated during certain behavioral categories of leg movements and scale with total movement speed. A previous study using extracellular single-unit recordings from brainstem serotonergic neurons in freely behaving cats found that a large proportion of cells exhibited increasing firing rates as locomotor speed increased (Veasey et al. 1995). Although only some serotonergic neurons in this region project to the spinal cord (Jacobs and Azmitia 1992) and it could not be confirmed that the recorded neurons were DNs, these results raise the idea that speed-dependent modulation of firing rates may be a common feature of many different populations of descending modulatory neurons, including the DA-DNs characterized in the current study.

Another important feature of the timing relationship between DA-DN activity and leg movements is that it provides insight into the source of movement-related activity in these cells. The movement-related activity observed in DA-DNs could originate centrally (i.e., inputs from other central brain neurons or corollary discharge from VNC motor circuits) or could be driven by peripheral sensory feedback (i.e., ascending inputs from leg sensory neurons encoding proprioceptive or mechanosensory feedback). We observed that the peak value of the cross-covariance between DA-DN spike rate and total leg speed occurs at a consistently negative time lag of ∽80 msec across experiments. This timing relationship indicates that, on average, increases in DA-DN spike rate precede increases in total leg speed, inconsistent with the idea that movement-related activity in DA-DNs is a consequence of peripheral sensory feedback. Thus, the activity in DA-DNs could, in principle, modulate leg movements with a short latency.

Given that left and right DA-DNs send strong projections to the ipsilateral side of the VNC, we anticipated that the activity of each DA-DN would be better related to the movements of legs ipsilateral to its cell body. In contrast, we found that DA-DN spike rates are equally well related to movements of the ipsilateral and contralateral legs, and furthermore, that DA-DN activity is also well related to the movements of the front, middle, and rear legs. One potential explanation for these findings is that movement-related activity in DA-DNs reflects synaptic inputs from neurons that specify descending commands for legs on both sides of the body. Indeed, there is a putative anatomical basis for this idea, because more dorsally situated DNs in the blowfly brain have been shown to collateralize extensively within the SEZ (Strausfeld et al. 1984), and we have identified clusters of DNs that reside in the dorsal protocerebrum in the *Drosophila* brain, some of which collateralize in the SEZ (data not shown). Although our data indicate that DA-DN movement-related activity is central in origin, additional studies will be required to determine the identity of the neurons that provide input to DA-DNs.

### Behavioral context-dependent recruitment of DA-DNs

Almost all transitions from tonic to burst firing in DA-DNs are associated with coincident leg movements. Importantly, the converse is not true; only a subset of leg movements are associated with bursts in DA-DNs. The conclusion that DA-DN bursts are recruited during only a subset of movements is further supported by the observation that increases in DA-DN spike rates were observed during kicking leg movements but not during rubbing. Previous studies in the locust have shown that subsets of VNC octopaminergic neurons are differentially recruited during fictive flight (Duch and Pflüger 1999), fictive walking (Baudoux et al. 1998), kicking (Burrows and Pflüger 1995), and stepping (Mentel et al. 2008). Our results support the idea that differential recruitment of modulatory neurons is a feature of motor control not only at the level of VNC but also at the level of descending neurons.

In contrast to their differential recruitment by behavioral category, DA-DN activity is similarly related to kicking leg movements whether they occur spontaneously or in response to presentation of a sensory stimulus. Thus, DA-DNs are recruited during certain categories of behavior, regardless of the sensory context in which movements are elicited. Additionally, DA-DNs failed to respond to the majority of sensory stimuli we presented to flies, and their activity is most consistently and strongly modulated during periods of leg movement. This was true even for sensory stimuli that consistently elicit robust leg movement responses, such as the attractive odor apple cider vinegar. Thus, DA-DNs do not simply act as detectors of rewarding or salient sensory events, as has been described for many other DA neurons (Schultz 2007; Bromberg-Martin et al. 2010; Waddell 2013). Even in the case of the mechanosensory stimulus that elicits sensory responses in DA-DNs, these sensory responses are not predictive of subsequent leg movements and add linearly with leg movement-related activity in the cells. DA-DNs are therefore quite different from other insect DNs that have been characterized, in which a sensory stimulus elicits neural activity that is then strongly coupled to initiation (Tanouye and Wyman 1980; Hedwig 2000; Fotowat et al. 2011; Kohatsu et al. 2011), modulation (Möhl and Bacon 1983; Severi et al. 2014), or inhibition (Perrins et al. 2002; Li et al. 2003) of motor output. Instead, our data suggest that DA-DN activity may be engaged during specific classes of behaviors, irrespective of the sensory context in which these behaviors occur.

### Effects of acute manipulations of DA-DN spike rate on ongoing leg movements

In contrast to findings in reduced body motor circuits preparations that treatment with dopamine receptor agonists elicits locomotor-like rhythms or limb movements (Claassen and Kammer 1986; Yellman et al. 1997; Tsyganov and Sakharov 2000; Puhl and Mesce 2008; Lapointe et al. 2009), we found that activation of DA-DNs in intact flies does not elicit leg movements. What factors can account for the discrepancy between the previous in vitro and the current in vivo findings? Bath application of dopaminergic receptor agonists in vitro likely results in high concentrations of drug present at numerous sites throughout the body motor circuit, simultaneously activating multiple cell types and multiple dopaminergic receptor types that may elicit complex and even competing effects on synaptic and intrinsic neuronal properties (Harris-Warrick et al. 1998). The circuit effects measured in vitro may therefore differ greatly from any behavioral consequences that result from the normal spatial and temporal pattern of dopaminergic inputs that occur during behavior in the intact animal. Based on our findings that increasing DA-DN activity does not elicit movement in intact flies and that movement onset often precedes burst spiking in DA-DNs, we favor the idea that DA-DNs act to modulate body motor circuits, rather than to activate them to initiate movements.

Despite the significant correlation between DA-DN spike rates and leg movement speed, we found that bidirectional manipulations of DA-DN activity had no effect on ongoing leg movements. Given the abundant evidence that the brain provides brakes on the output of body motor circuits that are absent in reduced preparations (M'Cracken 1907; McDaniel and Horsfall 1957; Brodfuehrer and Friesen 1986; Thompson 1986; Mullins and Friesen 2012), one interpretation of these data is that with all descending pathways intact, activation of DA-DNs is not sufficient to alter ongoing leg movements in the behavioral contexts we examined. A related possibility is that the effects of DA-DN inputs to downstream motor circuits must combine with changes in other neuromodulatory inputs to generate a detectable effect on leg movements, although it remains unknown whether and how other neuromodulatory neurons are recruited during leg movements in flies. In *Drosophila*, three octopaminergic neurons in the SEZ have been described that send projections to the VNC (Busch et al. 2009; Certel et al. 2010), and we have also observed a small number of serotonergic DNs (data not shown). Given that serotonin and octopamine, like dopamine, elicit locomotor-like outputs in a headless fly (Yellman et al. 1997) as well as in other body motor circuit preparations (Claassen and Kammer 1986; Tsyganov and Sakharov 2000; Jordan et al. 2008), an interesting possibility is that simultaneous manipulations of multiple descending or local VNC modulatory neuron populations are required to alter the moment-to-moment control of leg movements in intact animals (Miles and Sillar 2011; Sharples et al. 2014). Future studies should explore how the activity of DA-DNs and other populations of neuromodulatory neurons is related to leg movements in different sensory and behavioral contexts, and the preparation we have described in the current study is well suited to address these questions. In addition to potential effects on leg movements, it is possible that DA-DN activity might impact additional behavioral processes, such as wing movements, gut movements, or respiration, and future work should also examine these possibilities.

## Supporting Information

**Movie S1**. Confocal image stack of brain with representative DA-DN cell fill.

**Movie S2**. Confocal image stack of foreleg and midleg VNC with representative DA-DN cell fill.

**Movie S3**. Confocal image stack of midleg, hindleg, and abdominal VNC with representative DA-DN cell fill.

**Movie S4**. Video of a fly engaged in kicking leg movements.

**Movie S5**. Video of a fly engaged in rubbing leg movements.
